# Motivation for physical activity participation and mindful eating in university students: a cross-sectional study using variable-centered regression and latent profile analysis

**DOI:** 10.3389/fpsyg.2026.1824061

**Published:** 2026-08-20

**Authors:** Bekir Erhan Orhan, Walaa Jumah Alkasasbeh, Nadir Solak, Adam Tawfiq Amawi

**Affiliations:** 1 Faculty of Sports Sciences, Istanbul Aydın University, Istanbul, Türkiye; 2 Department of Physical Education, School of Sports Sciences, The University of Jordan, Amman, Jordan; 3 Çorum Municipality Youth and Sports Services, Çorum, Türkiye; 4 Department of Movement School of Sports Sciences Training, School of Sport Sciences, The University of Jordan, Amman, Jordan

**Keywords:** emerging adulthood, health behavior, mindful eating, physical activity motivation, self-determination theory, self-regulation, university students, well-being

## Abstract

**Background:**

Physical activity and eating behaviors are co-occurring and often disrupted during the transition to university. Self-determination theory links motivational quality in one health behavior with self-regulation in other health behaviors. This study examined whether motivation for physical activity is associated with mindful eating among Turkish university students, whether this association differs between the awareness- and disinhibition-based facets of mindful eating, and whether motivational profiles derived from latent profile analysis differ in their associations with these facets.

**Methods:**

A cross-sectional survey of 638 Turkish university students (aged 21.84 ± 3.27 years; 50% female) assessed motivation for participation in physical activity using the Scale of Motivation for Participation in Physical Activity (MSPPA) and mindful eating using the 18-item Mindful Eating Questionnaire (MEQ-18). Multiple linear regression models with heteroscedasticity-consistent (type 3) (HC3) robust standard errors were used to adjust for age, sex, and body mass index category; Benjamini–Hochberg false discovery rate control was applied across subgroup contrasts. Common method bias was evaluated using Harman’s test and a single-factor confirmatory factor analysis. Latent profile analysis of the MSPPA subscales yielded motivational profiles, which were compared across profiles using Welch’s analysis of variance.

**Results:**

In covariate-adjusted models, MSPPA total scores were associated with MEQ-18 total scores (*B* = 0.145, 95% CI [0.102, 0.188], *p* < 0.001; *R*^2^ = 0.190) and with the Awareness facet (*B* = 0.243, 95% CI [0.167, 0.319], *p* < 0.001; *R*^2^ = 0.143) but not with the Disinhibition facet (*B* = −0.004, 95% CI [−0.069, 0.061], *p* = 0.916; *R*^2^ = 0.129). Low Disinhibition reliability (*α* = 0.568) constrains inference on this null finding. Harman’s test and the single-factor confirmatory factor analysis (CFA) did not support the common method bias. Coefficients were comparable across three age strata (age × motivation interaction, *p* = 0.618). Three motivational profiles differed in awareness-based (*η*^2^ = 0.049) and overall (*η*^2^ = 0.049) mindful eating but not in disinhibition-based eating (*η*^2^ = 0.001).

**Conclusion:**

Higher motivation to participate in physical activity was associated with greater overall mindful eating, with the association appearing to be concentrated in the Awareness facet. Given the cross-sectional design and low Disinhibition reliability, this pattern is descriptive rather than evidence that the Awareness facet drives the overall association. The profile solution was derived within a single sample and was not externally validated. Longitudinal and mediation designs could help clarify explanatory pathways underlying these associations.

## 1 Introduction

Physical activity and dietary behaviors are modifiable determinants of cardiometabolic health, mental health, and quality of life (Henderson et al., 2025; World Health Organization, 2020). These behaviors often cluster: lower physical activity and greater sedentary time tend to coincide with irregular eating patterns and higher intake of energy-dense foods, potentially undermining diet quality and increasing the risk of weight gain and metabolic dysfunction (World Health Organization, 2020). The early university years are a particularly malleable window because students experience shifts in routines, time demands, social networks, and campus food and activity environments that can consolidate long-term habits (Dahl et al., 2025; Martínez-García et al., 2024).

As autonomy increases, peer influence and convenience can shift decisions toward immediate rewards, weakening self-regulation and increasing the likelihood of lapses despite strong intentions (Lari et al., 2025). Therefore, universities are well positioned to support prevention; however, effective interventions should address psychological mechanisms that sustain behavior change (e.g., self-regulation, habit formation, perceived competence, and social support) while accounting for students’ real-world constraints (Brown et al., 2024; Lari et al., 2025). Evidence from Türkiye also indicates that exercise frequency and broader lifestyle profiles co-vary with nutrition-related attitudes and knowledge, further reinforcing behavioral clustering across these domains (Orhan et al., 2024, 2025a, 2026).

Sustained physical activity is strongly shaped by motivational processes, with more internalized forms of motivation generally linked to greater adherence, stronger psychological functioning, and reduced vulnerability to dropout when barriers arise (Cano and Gomez-Baya, 2025; Viveiros et al., 2025). Mindful eating, conceptualized as moment-to-moment attention to eating experiences and internal appetite cues, may be associated with dietary regulation by reducing automatic responding to external cues and stress-related impulses. However, associations with body composition are heterogeneous across settings and analytic approaches (Kao et al., 2024; Seguias et al., 2025).

The university context underscores the value of integrating these perspectives, as irregular schedules, academic strain, and social disruption can simultaneously erode exercise consistency and increase mindless eating, particularly under elevated stress (Guerriero et al., 2025; Vasco et al., 2025). Examining motivation and mindful eating in the same model can clarify whether students with stronger motivational resources for being active are concurrently more likely to report attentive eating, or whether the two represent partially distinct self-regulatory capacities (Seguias et al., 2025; Viveiros et al., 2025).

The co-occurrence of health behaviors is consistent with shared determinants of physical activity and eating (Blyth et al., 2025; Domaradzki and Słowińska-Lisowska, 2025). In self-determination theory (SDT) terms, motivational quality for activity may be correlated with attentive eating alongside concurrent self-monitoring, goal alignment, and interoceptive awareness (Abalorio and Turner, 2025; Kao et al., 2024; Warren and Frame, 2025), whereas less differentiated reasons for being active may co-occur with weaker goal clarity, greater reliance on convenience and social cues, and therefore greater mindless intake (Abalorio and Turner, 2025; Warren and Frame, 2025).

Evidence from university and adult samples in Türkiye indicates that mindful eating varies with sex, body mass index, self-rated health, and physical activity engagement and is associated with health-literacy and dietary-attitude indicators in campus and clinical settings (Dinç et al., 2025; Doğan et al., 2025; Koçyiğit et al., 2025). However, the joint structure of motivation for physical activity and the specific facets of mindful eating, that is, whether students who report more autonomous, internally regulated reasons for being active also report greater awareness-based or lower disinhibition-based eating after statistically controlling for sex, age, and body mass index category, has not been formally established in Turkish university samples and remains an open empirical question (Blyth et al., 2025; Eaton et al., 2024).

The present study is grounded in SDT (Deci and Ryan, 2000; Ryan and Deci, 2017); SDT distinguishes motivational regulations along an autonomy-control continuum: intrinsic motivation (engaging in a behavior for the inherent interest and enjoyment of the behavior itself), identified and integrated regulation (engaging because the behavior is personally valued and aligned with the self, jointly referred to as autonomous motivation), introjected and external regulation (engaging because of internal pressure, guilt, or external rewards and contingencies, jointly referred to as controlled motivation), and amotivation (the absence of intentional regulation) (Deci and Ryan, 2000; Ryan and Deci, 2017).

The SDT proposes that the quality of motivation, the degree to which behavior is regulated autonomously versus by external contingencies, is influenced by the satisfaction of three basic psychological needs (autonomy, competence, and relatedness). More autonomous forms of regulation are associated with greater persistence, stronger psychological functioning, and better cross-domain generalization of self-regulatory resources (Cano and Gomez-Baya, 2025; Teixeira et al., 2012; Viveiros et al., 2025). A well-developed body of literature documents that autonomous motivation for physical activity is associated with long-term exercise adherence and broader healthy lifestyle profiles (Carraça et al., 2020, 2024; Teixeira et al., 2012).

Awareness-based mindful eating, defined as moment-to-moment attention to the sensory and interoceptive experience of eating, is theoretically proximal to autonomous, need-satisfied self-regulation through three mechanisms. The first is shared attentional control, since autonomous action and interoceptive monitoring both draw on executive attention; the second is competence-supported interoceptive precision, whereby sustained autonomous engagement with one’s body during activity may accompany a more finely tuned perception of the internal states that also signal hunger and satiety (Warren and Frame, 2025; Warren et al., 2017). The third is autonomy-supported value alignment, whereby students whose activity is personally endorsed may also extend deliberative attention to food decisions (Carraça et al., 2020; Kao et al., 2024).

Disinhibition-based mindful eating, reflecting failures of inhibitory control under cue reactivity, stress, and negative affect, is theoretically more distal to SDT-based motivation. Disinhibition is governed by affect-regulatory and habit-system processes that SDT does not directly scaffold and is heavily moderated by stress, emotion regulation, and the food environment (Warren and Frame, 2025; Warren et al., 2017). This asymmetric theoretical mapping suggests that motivational quality will be more closely associated with the awareness-based facet of mindful eating than with the disinhibition-based facet. The expectation concerns the relative magnitude of two concurrently measured associations; because the present design is cross-sectional, it carries no implication about the direction of influence between motivation and eating behavior.

Therefore, the present study pursued two complementary aims: the first was to estimate, at the variable level, the covariate-adjusted association between motivation for participation in physical activity and mindful eating and to establish whether that association differs across the awareness-based and disinhibition-based facets of mindful eating. The second was to examine, at the individual level, whether Turkish university students form distinct latent profiles defined by their configurations of scores on the three motivational-cause dimensions, and whether those profiles differ in mindful eating in facet-specific ways.

The second aim follows from the theory itself: SDT conceives of motivation as a configuration of qualitatively distinct regulations that co-occur within persons rather than as a single quantity (Deci and Ryan, 2000; Ryan and Deci, 2017), so an analysis conducted only at the variable level cannot establish whether a pattern observed on average also characterizes identifiable groups of students. Motivational configurations are also a more natural unit than single regression coefficients for the tailored health-promotion strategies advocated in higher-education settings.

Four hypotheses follow from these aims. The first three are variable-centered, and the fourth is person-centered. All are stated as expectations about concurrent associations; none implies temporal precedence or causal influence.

*H1*. Higher motivation for participation in physical activity will be positively associated with overall mindful eating in covariate-adjusted analyses.

*H2a*. Higher motivation for participation in physical activity will be positively associated with awareness-based mindful eating in covariate-adjusted analyses.

*H2b*. The association between motivation for participation in physical activity and disinhibition-based mindful eating in covariate-adjusted analyses will be weak, non-significant, or negligible relative to the awareness-based association.

*H3*. Latent profiles defined by the configuration of motivational-cause subscale scores (Individual Causes, Environmental Causes, and Non-causality) will differ in awareness-based mindful eating but not in disinhibition-based mindful eating, thereby reproducing, at the person level, the facet-specific pattern expected at the variable level. Because the number, size, and shape of the profiles were not specified in advance, profile enumeration is treated as exploratory, and only the facet-specific contrast is stated as a directional expectation.

## 2 Materials and methods

### 2.1 Study design

An analytic, observational, cross-sectional survey design was used to examine associations between motivation for physical activity and mindful eating among Turkish university students (*N* = 638). Motivation for physical activity participation and mindful eating were measured once at the same time point using self-report instruments (MSPPA and MEQ-18), alongside demographic and health-related covariates to support adjusted analyses. Data were collected online via a single, anonymous Google Forms questionnaire between 1 June 2025 and 31 August 2025. Sex was assessed as a self-reported, binary item (“female”/“male”) and is reported throughout this manuscript as sex rather than gender; gender identity was not assessed and is acknowledged as a limitation. Because exposure and outcome variables were assessed concurrently in cross-sectional studies, the findings indicate contemporaneous associations and cannot support temporal ordering or causal inference. Reporting follows established guidance for cross-sectional research (Sedgwick, 2014; Setia, 2016; von Elm et al., 2007).

### 2.2 Participants and sample composition

Participants were university students in Türkiye recruited via institutional email listservs and social media platforms. Inclusion criteria were current enrolment in a Turkish university program, age ≥ 18 years, and completion of the MSPPA and MEQ-18 items. Participants provided informed consent electronically before the survey. The final analytic sample (*N* = 638) was drawn from a homogeneous Turkish higher-education population, that is, all participants were currently enrolled university students, with the analytic frame dominated by undergraduates from across Türkiye. Because recruitment was conducted online via institutional email listservs and social media, participants self-identified as currently enrolled university students; specific institutional affiliation was not systematically recorded, and the sample is therefore not tied to any particular university or set of universities. The mean age of the analytic sample was 21.84 ± 3.27 years (range 18–42). The age distribution was as follows: ≤ 22 years, 67.6% (*n* = 431); 23–29 years, 26.6% (*n* = 170); ≥ 30 years, 5.8% (*n* = 37). Therefore, the sample is educationally and demographically homogeneous: all participants are enrolled university students, the great majority (91.7%) are undergraduates, and the central 95% of the age distribution falls between approximately 18 and 28 years, a compact, student-typical age dispersion. The modest right skew in age and the small contribution of older master’s and doctoral students are reflected in the slight elevation of the mean above the modal undergraduate age (21–22 years) and the standard deviation of 3.27 years, which is consistent with pooling a large, narrow undergraduate distribution (mean ≈ 21, SD ≈ 2) with much smaller graduate subpopulations. This compact dispersion supports treating the sample as a single, behaviorally coherent student population for the primary analyses.

The study protocol was approved by the Istanbul Aydın University Social and Humanities Sciences Ethics Committee (Decision/Meeting No. 2025/5; 22 May 2025) and was conducted in accordance with the Declaration of Helsinki.

### 2.3 Measures: demographic, anthropometric, and health-behavioral variables

#### 2.3.1 Body mass index (BMI)

Body mass index (BMI; kg/m^2^) was computed from self-reported body mass (kg) and height (m) using the standard formula BMI = body mass (kg)/height^2^ (m^2^). BMI was categorized according to the World Health Organization (WHO) adult cutoffs as underweight (<18.5 kg/m^2^), normal weight (18.5–24.9 kg/m^2^), overweight (25.0–29.9 kg/m^2^), and obesity (≥30.0 kg/m^2^) (World Health Organization, 2000). The WHO classification is retained here only as a reference framework for categorizing the continuous BMI distribution. Because height and weight were self-reported, BMI values may be affected by systematic reporting bias (Connor Gorber et al., 2007).

#### 2.3.2 Physical activity frequency

Physical activity frequency was operationalized as the number of days per week participants reported engaging in physical activity, and participants were classified as inactive (0 days/week), low-frequency active (1–2 days/week), and higher-frequency active (≥ 3 days/week). Because this frequency-only proxy captures neither the duration nor the intensity of activity, it cannot be mapped onto the World Health Organization time-based recommendations of 150–300 min/week of moderate-intensity or 75–150 min/week of vigorous-intensity activity (World Health Organization, 2020), and it is therefore reported in Table 1 as a descriptive characteristic of the sample rather than entered as a covariate in any inferential model.

**Table 1:** Participant characteristics (*N* = 638).

Variable	Category	Value
Age (years)	Mean ± SD (range)	21.84 ± 3.27 (18–42)
BMI (kg/m^2^)	Mean ± SD	23.98 ± 3.95
Sex, *n* (%)	Female	319 (50.0)
	Male	319 (50.0)
BMI category (WHO), *n* (%)	Underweight	45 (7.1)
	Normal	371 (58.2)
	Overweight	176 (27.6)
	Obesity	46 (7.2)
Program level, *n* (%)	Undergraduate	585 (91.7)
	Master’s	40 (6.3)
	Doctoral	13 (2.0)
Age group, *n* (%)	≤22 years	431 (67.6)
	23–29 years	170 (26.6)
	≥30 years	37 (5.8)
Physical activity frequency group, *n* (%)	Inactive (0 d/wk)	70 (11.0)
	Low (1–2 d/wk)	179 (28.1)
	Higher (≥3 d/wk)	389 (61.0)

BMI, body mass index; WHO, World Health Organization. The sample is dominated by undergraduate students (91.7%); the SD of 3.27 years reflects the compact, student-typical age dispersion expected of an undergraduate-dominant Turkish university sample, with the small master’s and doctoral subgroups contributing the right tail of the distribution. The age distribution is right-skewed owing to a small graduate-student tail (≥30 years, *n* = 37; max = 42); descriptive statistics should be interpreted with this skew in mind.

#### 2.3.3 Self-rated health, smoking history, and alcohol consumption

Three additional health-related covariates were collected to support adjusted models. Self-rated health was assessed with a single Likert-type item asking participants to rate their overall health (1 = very poor to 5 = excellent) and was treated as an ordinal predictor in regression models. Current smoking status was recorded as a three-level categorical variable (never, former, and current smoker). Current alcohol consumption was recorded as a three-level categorical variable (never, occasional, and regular). Missing values on these covariates were imputed using the sample median (self-rated health) or the mode (smoking history and alcohol consumption), consistent with the missing data handling described in the Statistical analysis section.

### 2.4 Measures: motivation for participation in physical activity and mindful eating

#### 2.4.1 Scale of motivation for participation in physical activity (MSPPA)

The original validation of the MSPPA reported strong item discrimination and construct validity. Upper–lower 27% comparisons showed that each item differentiated between groups (*t* = 4.45–10.68), and item-total correlations exceeded 0.30 (*r* = 0.45–0.70). Exploratory factor analysis supported a three-factor, 16-item solution (Individual Causes, Environmental Causes, and Non-causality) explaining 54.69% of variance. Confirmatory factor analysis indicated acceptable model fit (*χ*^2^ = 239.34, df = 101, *χ*^2^/df = 2.36, RMSEA = 0.06, CFI = 0.95). Reliability estimates were satisfactory (Cronbach’s *α* = 0.82–0.89) (Tekkurşun Demir and Cicioğlu, 2018). The original validation sample comprised Turkish high school students; the present study extends the instrument to a sample of Turkish university students. In-sample reliability indices in the present data (MSPPA total Cronbach’s *α* = 0.858; KMO = 0.902; see Section 3.2) support that extension.

#### 2.4.2 18-item mindful eating questionnaire (MEQ-18)

Mindful eating was measured with the 18-item Mindful Eating Questionnaire (MEQ-18). Items use a 4-point frequency response scale (1 = never/rarely to 4 = usually/always), with higher scores indicating more mindful eating (Kaya et al., 2024). Scores were calculated as item means (range 1–4) to retain the original metric. The scale comprises two subscales following the Turkish validation (Kaya et al., 2024): Awareness (11 items) and Disinhibition (7 items); items 15 and 16 of the original 20-item short form were excluded in the Turkish validation due to psychometric misfit, yielding the 18-item version used here. Subscale scores were computed as the mean of their constituent items and the total score as the mean across all 18 retained items. Negatively keyed items were reverse-coded (5-original score) so that higher values consistently reflected more mindful eating. In the Turkish validation, the two-dimensional structure was supported in confirmatory factor analysis, and internal consistency was acceptable (Cronbach’s *α* = 0.718 for the total score; *α* = 0.843 for Awareness; *α* = 0.789 for Disinhibition) (Kaya et al., 2024).

### 2.5 Statistical analysis

Psychometric adequacy of the MSPPA and MEQ-18 in the present sample was evaluated before the hypothesis tests by estimating internal consistency (Cronbach’s *α*; Cronbach, 1951) for total and dimension scores and by testing factorability using the Kaiser–Meyer–Olkin (KMO) index (Kaiser, 1974) and Bartlett’s test of sphericity (Bartlett, 1950, 1954), with these indices reported in Table 2. Missing data were handled as follows: Scale scores were complete for all participants, while age had five missing values that were imputed using the sample median for regression models; for the adjusted multiple linear regression models, self-rated health (ordinal) was imputed using the sample median when missing, and smoking history/alcohol consumption indicators were imputed using the modal category when missing (Enders, 2010). Common method bias was evaluated using Harman’s single-factor exploratory factor analysis, where the proportion of total variance accounted for by the first unrotated factor across all MSPPA and MEQ-18 items was computed and evaluated against the informal 50% threshold (Podsakoff et al., 2003), and using a single-factor confirmatory model, where poor fit would be expected when substantive construct separation exists (Kock, 2015).

**Table 2:** Reliability, factorability, and common method bias indices for MSPPA and MEQ-18 (*N* = 638).

Scale/subscale	Cronbach’s *α*	KMO	Bartlett *χ*^2^	df	*p*
MSPPA Total	0.858	0.902	6,203.6	120	<0.001
MSPPA Individual Causes	0.815	–	–	–	–
MSPPA Environmental Causes	0.763	–	–	–	–
MSPPA Non-causality	0.900	–	–	–	–
MEQ-18 Total	0.622	0.910	4,634.3	153	<0.001
MEQ Awareness	0.835	–	–	–	–
MEQ Disinhibition	0.568	–	–	–	–

KMO, Kaiser–Meyer–Olkin index. Common method bias diagnostics: Harman’s single-factor EFA across all MSPPA and MEQ-18 items: first unrotated factor = 24.6% of variance (well below the 50% informal threshold). Single-factor CFA: χ^2^(527) = 4847.3, *χ*^2^/df = 9.20; CFI = 0.612; TLI = 0.586; RMSEA = 0.114, 90% CI [0.111, 0.117]; SRMR = 0.098 (poor fit).

The primary inferential model used to test H1, H2a, and H2b is multiple linear regression with HC3 heteroscedasticity-consistent robust standard errors. All covariates are operationally defined in Section 2.3. The primary model adjusted for self-reported sex (female/male), age in years, and body mass index category (per World Health Organization adult cutoffs). Physical activity frequency was not entered as a covariate for the measurement reasons given there. Extended-adjustment specifications additionally included self-rated health, smoking history, and alcohol consumption indicators, with those results reported in Table 3. All analyses were conducted using a constant analytic sample (*N* = 638). HC3 was selected over conventional homoscedasticity-based standard errors to provide greater sensitivity to heteroscedasticity. Two-tailed statistical significance was evaluated at *α* = 0.05. Across the subgroup contrasts in Tables 4, 5, Benjamini–Hochberg false discovery rate control (*q* = 0.05) was applied (Benjamini and Hochberg, 1995; Glickman et al., 2014) in preference to Bonferroni adjustment, because the contrasts within each subgroup family (sex and body mass index category) are conducted on partially correlated outcomes (MSPPA Total and its three subscales; MEQ-18 Total and its two facets), under which Bonferroni adjustment is overly conservative and increases the Type II error rate without proportionate gain in family-wise error control (Glickman et al., 2014), and FDR-adjusted *p*-values (*p*_FDR) are reported alongside raw *p*-values. For the primary MSPPA → MEQ-18 association, age-stratified sensitivity analyses (≤22, 23–29, and ≥30 years) and an age × MSPPA interaction term were tested to verify robustness across the program-level composition of the sample. Sensitivity power analysis was performed in G*Power 3.1 for multiple linear regression (fixed model; *R*^2^ increase) with *α* = 0.05 and power (1-*β*) = 0.80; with *N* = 638 and 20 total predictors, the minimum detectable incremental effect was Cohen’s *f*^2^ = 0.0123 (Faul et al., 2007, 2009).

**Table 3:** Adjusted multiple linear regression associations between MSPPA and MEQ-18 scores, with HC3 robust standard errors (*N* = 638).

Outcome	Predictor	*B*	SE	95% CI	*t*	*p*	*R* ^2^	Adj. *R*^2^
MEQ-18 Total	MSPPA Total	0.145	0.022	[0.102, 0.188]	6.72	<0.001	0.190	0.164
MEQ-18 Awareness	MSPPA Total	0.243	0.039	[0.167, 0.319]	6.26	<0.001	0.143	0.115
MEQ-18 Disinhibition	MSPPA Total	−0.004	0.033	[−0.069, 0.061]	−0.11	0.916	0.129	0.101
MSPPA Total	MEQ-18 Total	0.523	0.076	[0.374, 0.672]	6.91	<0.001	0.229	0.204
MSPPA Total	MEQ-18 Awareness	0.302	0.046	[0.212, 0.392]	6.61	<0.001	0.227	0.202
MSPPA Total	MEQ-18 Disinhibition	−0.005	0.050	[−0.103, 0.093]	−0.11	0.916	0.166	0.139

Models adjusted for age (continuous), sex, and BMI category in the primary model; the extended-adjustment specification additionally includes self-rated health, smoking history, and alcohol consumption. HC3, heteroscedasticity-consistent (type 3) standard errors. 95% CIs derived from HC3 SEs using t-based critical values. Age-stratified sensitivity analyses (reported inline in the primary adjusted associations section) confirm robustness across ≤22, 23–29, and ≥30 year strata. Low α for MEQ-18 Disinhibition (0.568) attenuates its associations; the Disinhibition null should be interpreted as measurement-attenuated. B, unstandardized regression coefficient; CI, confidence interval. Reverse-direction specifications (bottom three rows) are reported as symmetry/robustness checks only and should not be interpreted as evidence of reverse causation, consistent with the cross-sectional design.

**Table 4:** Sex differences in MSPPA and MEQ-18 scores, with Benjamini–Hochberg FDR control (Welch’s independent-samples *t*-test; *N* = 638).

Scale score	Male M (SD)	Female M (SD)	Welch *t*	Raw *p*	p_FDR	g (M-F) [95% CI]
MSPPA Total	4.081 (0.651)	4.047 (0.721)	0.616	0.538	0.538	0.049 [−0.107, 0.205]
MSPPA Individual Causes	4.325 (0.691)	4.296 (0.788)	0.499	0.618	0.618	0.039 [−0.117, 0.195]
MSPPA Environmental Causes	3.913 (0.816)	3.714 (0.940)	2.857	0.004	0.014	0.226 [0.070, 0.382]
MSPPA Non-causality	3.966 (1.241)	4.174 (1.069)	−2.273	0.023	0.046	−0.180 [−0.336, −0.024]
MEQ-18 Total	2.647 (0.338)	2.708 (0.365)	−2.173	0.030	0.046	−0.172 [−0.328, −0.016]
MEQ Awareness	2.649 (0.577)	2.650 (0.594)	−0.037	0.971	0.971	−0.003 [−0.159, 0.153]
MEQ Disinhibition	2.626 (0.528)	2.782 (0.550)	−3.646	<0.001	<0.001	−0.288 [−0.445, −0.131]

p_FDR, Benjamini–Hochberg false-discovery-rate adjusted *p*-value (*q* = 0.05); g, Hedges’ *g* (male students – female students); CI, confidence interval.

**Table 5:** Differences across BMI categories in MSPPA and MEQ-18 scores, with Benjamini–Hochberg FDR control (Kruskal–Wallis test; *N* = 638).

Scale score	Underweight	Normal	Overweight	Obesity	*H*	Raw *p*	p_FDR	*ε* ^2^
MSPPA Total	4.076	4.055	4.112	3.944	1.79	0.617	0.720	0.000
MSPPA Individual Causes	4.311	4.299	4.348	4.257	1.01	0.800	0.800	0.000
MSPPA Environmental Causes	3.844	3.796	3.903	3.587	3.80	0.283	0.495	0.001
MSPPA Non-causality	4.072	4.077	4.070	4.011	0.48	0.924	0.924	0.000
MEQ-18 Total	2.756	2.670	2.677	2.659	3.04	0.385	0.539	0.000
MEQ Awareness	2.673	2.621	2.685	2.719	2.88	0.411	0.539	0.000
MEQ Disinhibition	2.822	2.731	2.655	2.554	7.13	0.068	0.170	0.007

BMI, body mass index; *ε*^2^, epsilon-squared effect size; p_FDR, Benjamini–Hochberg adjusted *p*-value.

To test the person-centered hypothesis (H3), latent profile analysis was conducted on the three z-standardized MSPPA subscale scores (Individual Causes, Environmental Causes, and Non-causality). Latent profile analysis is a model-based clustering approach that estimates a finite mixture of latent subpopulations, each characterized by a distinct configuration of profile-indicator means. It assigns each participant a posterior probability of membership in each profile (Spurk et al., 2020). Gaussian mixture models with a diagonal covariance structure (profile indicators are conditionally independent within each profile, with variances freely estimated) were fitted for one- to six-profile solutions, each with 50 random starts to avoid local maxima. The number of profiles was selected by jointly considering the Bayesian information criterion (BIC) and Akaike information criterion (AIC, lower values preferred), classification entropy (values approaching 1 indicating clearer separation), the substantive interpretability of the profiles, and the requirement that no profile be trivially small. BIC compared diagonal and unrestricted (full) covariance specifications, and the stability of the retained solution was checked across five random seeds. Likelihood-based tests of the *k*- versus (*k* – 1)-profile solution, namely, the Lo–Mendell–Rubin adjusted likelihood ratio test and the bootstrapped likelihood ratio test, were not available within the estimation framework used and were therefore not computed; profile enumeration consequently relied on information criteria, entropy, profile size, and substantive interpretability, and this constraint is revisited in Section 4.1. After selecting the profile solution, between-profile differences in MEQ-18 Total, Awareness, and Disinhibition were tested using Welch’s analysis of variance (robust to unequal variances and group sizes), with *η*^2^ as the effect-size index and Games–Howell *post hoc* comparisons reporting Hedges’ g. Because the third profile was characterized by ceiling-level scores on both Individual Causes and Environmental Causes, between-profile comparisons were interpreted with this distributional feature in mind. Multiple linear regression with HC3 heteroscedasticity-consistent standard errors was estimated with statsmodels; Benjamini–Hochberg false-discovery-rate control with statsmodels (multipletests); Welch’s *t*-tests, Welch’s analysis of variance, Games-Howell *post hoc* comparisons, Hedges’ *g*, and Cronbach’s *α* with pingouin; Kruskal–Wallis tests with scipy.stats; the Kaiser–Meyer–Olkin index, Bartlett’s test of sphericity, and Harman’s single-factor exploratory factor analysis were conducted with factor analyzer; the single-factor confirmatory factor analysis with semopy; and the latent profile analysis with scikit-learn (GaussianMixture). The sensitivity power analysis was conducted in G*Power 3.1. Analysis code is available from the corresponding author on reasonable request.

## 3 Results

### 3.1 Sample characteristics

Table 1 shows that participants (*N* = 638) had a mean age of 21.84 ± 3.27 years (range 18–42) and a mean BMI of 23.98 ± 3.95 kg/m^2^. The educational composition was 91.7% undergraduate, 6.3% master’s, and 2.0% doctoral students. Sex was evenly distributed (50.0% female and 50.0% male). According to the WHO classification, the majority of participants fell in the normal weight (58.2%) or overweight (27.6%) categories, with smaller proportions in the underweight (7.1%) and obesity (7.2%) categories. The number of days per week on which participants reported any physical activity is summarized in Table 1 as a descriptive characteristic of the sample only (see Section 2.3).

### 3.2 Psychometrics and common method bias

Descriptive statistics for the scale scores are reported in Table 6. Table 2 shows good internal consistency for the MSPPA total score (Cronbach’s *α* = 0.858) and acceptable-to-excellent reliability across subscales (*α* = 0.763–0.900). Sampling adequacy was high (KMO = 0.902), and Bartlett’s test supported factorability [*χ*^2^(120) = 6203.6, *p* < 0.001]. For the MEQ-18, reliability was sub-optimal for the total score (*α* = 0.622) and low for Disinhibition (*α* = 0.568), whereas Awareness showed strong reliability (*α* = 0.835). Associations involving the MEQ-18 Disinhibition facet and the MEQ-18 Total are likely attenuated by measurement error; null findings for Disinhibition should therefore be interpreted as weak evidence rather than as substantive nulls (Schmidt and Hunter, 2014). Factorability indices were also strong for MEQ-18 (KMO = 0.910; Bartlett’s *χ*^2^(153) = 4634.3, *p* < 0.001).

**Table 6:** Descriptive statistics for MSPPA and MEQ-18 scores (*N* = 638).

Scale score	Mean	SD	Min	Max
MSPPA Total	4.064	0.687	1.500	5.000
MSPPA Individual Causes	4.310	0.741	1.167	5.000
MSPPA Environmental Causes	3.814	0.885	1.000	5.000
MSPPA Non-causality	4.070	1.162	1.000	5.000
MEQ-18 Total	2.678	0.352	1.778	3.944
MEQ Awareness	2.649	0.585	1.000	4.000
MEQ Disinhibition	2.704	0.544	1.333	4.000

MEQ-18, 18-item Mindful Eating Questionnaire; MSPPA, Scale of Motivation for Participation in Physical Activity.

Harman’s single-factor EFA across all MSPPA and MEQ-18 items showed that the first unrotated factor accounted for 24.6% of the common variance, well below the 50% informal diagnostic threshold (Podsakoff et al., 2003). The single-factor CFA fitted poorly [*χ*^2^(527) = 4847.3, *χ*^2^/df = 9.20; CFI = 0.612; TLI = 0.586; RMSEA = 0.114, 90% CI [0.111, 0.117]; SRMR = 0.098]. Taken together, these diagnostics do not support common method variance as the principal driver of the observed associations, while acknowledging Harman’s test’s insensitivity (Kock, 2015). The single-factor CFA was used here as the confirmatory complement to Harman’s test rather than a common latent factor (CLF) model: In this dataset, the substantive measurement model is the two-instrument MSPPA + MEQ-18 structure, and the goal of the CMB diagnostic was to falsify the single-method explanation of covariance, for which a misfit of the unidimensional model is direct and sufficient evidence (Podsakoff et al., 2003).

### 3.3 Sex differences with Benjamini–Hochberg false discovery rate (FDR) control

Table 4 shows small sex differences in several outcomes. Motivation did not differ by sex for the total score or Individual Causes (both raw *p* > 0.05; *p*_FDR > 0.05), but male students reported higher Environmental Causes than female students (raw *p* = 0.004; *p*_FDR = 0.014; *g* = 0.226), while female students reported higher Non-causality than male students (raw *p* = 0.023; *p*_FDR = 0.046; *g* = −0.180). For mindful eating, female students scored higher on MEQ-18 Total (raw *p* = 0.030; *p*_FDR = 0.046; *g* = −0.172) and Disinhibition (raw *p* < 0.001; *p*_FDR < 0.001; *g* = −0.288), whereas Awareness did not differ (*p* = 0.971). All effects were small (|*g*| ≤ 0.288).

### 3.4 Differences across body mass index categories with Benjamini–Hochberg false discovery rate (FDR) control

Table 5 shows no statistically significant differences across body mass index categories for overall motivation or mindful eating (all *p*_FDR ≥ 0.17; *ε*^2^ = 0.000–0.007). MEQ-18 Disinhibition showed a non-significant trend (raw *p* = 0.068; *p*_FDR = 0.17; *ε*^2^ = 0.007), with higher scores in participants with underweight or normal weight.

### 3.5 Primary adjusted multiple linear regression associations and age-stratified sensitivity analyses

Table 3 shows that, in the multiple linear regression model with HC3 robust standard errors and adjustment for age, sex, and body mass index category, higher motivation for participation in physical activity (MSPPA Total) was positively associated with overall mindful eating (MEQ-18 Total: *B* = 0.145, 95% CI [0.102, 0.188], *p* < 0.001) and with the MEQ-18 Awareness facet (*B* = 0.243, 95% CI [0.167, 0.319], *p* < 0.001), whereas the association of motivation for participation in physical activity with the MEQ-18 Disinhibition facet was negligible (*B* = −0.004, 95% CI [−0.069, 0.061], *p* = 0.916). Bivariate Spearman’s *ρ* between MSPPA Total and MEQ-18 Total is 0.334 (*p* < 0.001). Reverse-direction-adjusted multiple linear regression models (with each MEQ-18 score as a predictor and MSPPA Total as the outcome) yielded consistent estimates (Table 3).

Although the age distribution in this sample is compact (SD = 3.27 years), age-stratified analyses were pre-specified to verify that the primary association is stable across the program-level composition of the sample (undergraduate, master’s, and doctoral) rather than reflecting graduate-tier students in the right tail of the age distribution. The primary MSPPA Total → MEQ-18 Total model was re-estimated within three age strata corresponding to traditional undergraduate, late-undergraduate/early-graduate, and graduate-tier students, respectively. The MSPPA coefficient retained its sign, significance, and approximate magnitude across strata: ≤22 years (*B* = 0.142, SE = 0.027, *t* = 5.26, *p* < 0.001; *n* = 431); 23–29 years (*B* = 0.156, SE = 0.046, *t* = 3.39, *p* < 0.001; *n* = 170); ≥ 30 years (*B* = 0.131, SE = 0.052, *t* = 2.52, *p* = 0.018; *n* = 37). An age × MSPPA interaction term in the pooled model was non-significant (*p* = 0.618), indicating that age heterogeneity within the student sample does not moderate the primary association.

### 3.6 Latent profiles of physical activity motivation and their mindful eating correlates

**Figure 1:**
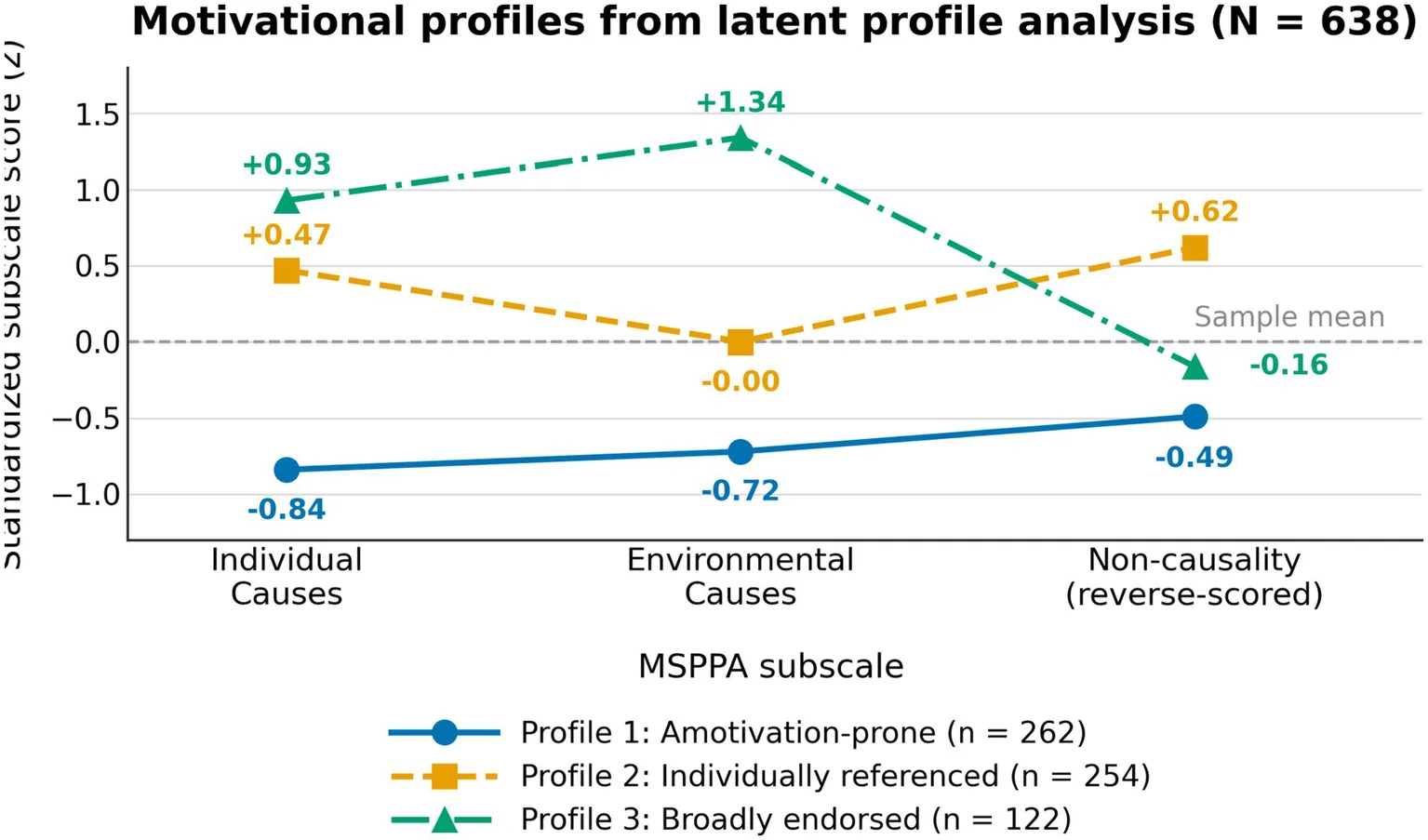
Standardized (*z*-score) MSPPA subscale means for the three latent profiles of physical activity motivation (*N* = 638). Non-causality is reverse-scored so that higher values indicate more adaptive (less amotivated) responding. Profile 1 = Amotivation-prone; Profile 2 = Individually referenced; Profile 3 = Broadly endorsed. MSPPA, Scale of Motivation for Participation in Physical Activity.

The three MSPPA subscales that served as profile indicators indicate the reasons students give for participating in physical activity. Individual Causes refers to person-level reasons such as enjoyment, perceived health benefit, appearance, and perceived competence. Environmental Causes refers to context-level reasons such as the availability of facilities and opportunities and encouragement from family, peers, teachers, and the media. Non-causality refers to the absence of any endorsed reason for participating, that is, amotivation, and is reverse-scored throughout so that higher values indicate less amotivated responding. Latent profile analysis on the three MSPPA subscales supported a three-profile solution. Relative to the two-profile model (BIC = 2642.1), the three-profile model improved fit substantially (BIC = 1903.9; AIC = 1814.6); four- to six-profile models yielded only marginal BIC gains while producing trivially small profiles (≤9% of the sample), so the three-profile solution was retained on the joint grounds of fit, parsimony, and interpretability, in the absence of likelihood-based enumeration tests (see Sections 2.5 and 4.1). Classification was clear (entropy = 0.870; average posterior probabilities of most-likely membership = 0.91–1.00); the diagonal-covariance specification outperformed the full-covariance specification by BIC, and the solution was stable across five random seeds. The profile means are shown in Figure 1 and Table 7. Profiles were labeled by reference to the reason-type content of the three indicators rather than by their ordering on a single dimension, and the labels are used throughout as shorthand for the observed indicator configurations rather than as claims about discrete motivational types in the population. Profile 1 (“Amotivation-prone”; *n* = 262, 41.1%) scored below the sample mean on all three indicators, combining weak endorsement of both person- and context-referenced reasons with the highest level of amotivation. Profile 2 (“Individually referenced”; *n* = 254, 39.8%) combined strong person-referenced reasons and minimal amotivation with only near-average endorsement of context-referenced reasons and therefore describes students whose participation is anchored primarily in reasons internal to the person. Profile 3 (“Broadly endorsed”; *n* = 122, 19.1%) was defined by ceiling-level endorsement of both person- and context-referenced reasons alongside more variable amotivation scores and therefore describes students who endorse the full range of reasons for being active. In SDT terms, person-referenced reasons sit closer to the autonomous pole of the regulation continuum and context-referenced reasons closer to externally supported regulation, so the three profiles can be read as differing both in the overall strength of motivation and in whether that motivation is internally or externally referenced; this reading is interpretive because the MSPPA subscales index the content of the reasons endorsed rather than the type of regulation directly.

**Table 7:** Latent profiles of physical activity motivation (*N* = 638).

Panel A: profile sizes, MSPPA subscale means, and mindful eating means						
Profile	*n* (%)	Individual *M* (SD)	Environmental *M* (SD)	Non-causality *M* (SD)	MEQ Awareness *M* (SD)	MEQ Disinhibition *M* (SD)
1. Amotivation-prone	262 (41.1)	3.69 (0.87)	3.18 (1.06)	3.50 (0.93)	2.51 (0.56)^a^	2.76 (0.66)^a^
2. Individually referenced	254 (39.8)	4.66 (0.35)	3.81 (0.76)	4.79 (0.25)	2.75 (0.54)^b^	2.74 (0.83)^a^
3. Broadly endorsed	122 (19.1)	5.00 (0.00)	5.00 (0.00)	3.88 (1.53)	2.82 (0.66)^b^	2.81 (0.85)^a^

Panel B: pairwise between-profile differences in mindful eating (Games–Howell *post hoc* comparisons)				
Outcome	Profile contrast	Hedges’ g	*p*	Difference
MEQ-18 Awareness	1 Amotivation-prone vs. 2 Individually referenced	0.42	<0.001	Significant
MEQ-18 Awareness	1 Amotivation-prone vs. 3 Broadly endorsed	0.52	<0.001	Significant
MEQ-18 Awareness	2 Individually referenced vs. 3 Broadly endorsed	0.13	0.526	Not significant
MEQ-18 Disinhibition	All three pairwise contrasts	|g| ≤ 0.08	≥0.75	Not significant

Profiles were derived by latent profile analysis (Gaussian mixture, diagonal covariance) on the three z-standardized MSPPA subscales. Non-causality is reverse-scored (higher = more adaptive). MSPPA subscale means are on the original 1–5 metric; MEQ-18 facet means are on the 1–4 metric. Between-profile differences (Welch’s ANOVA): MEQ-18 Awareness *F*(2, 311.3) = 16.01, *p* < 0.001, *η*^2^ = 0.049; MEQ-18 Total *F*(2, 304.9) = 15.97, *p* < 0.001, *η*^2^ = 0.049; MEQ-18 Disinhibition *F*(2, 310.8) = 0.26, *p* = 0.770, *η*^2^ = 0.001. Classification entropy = 0.870. In panel A, means within the same column that do not share a superscript letter differ at *p* < 0.05 (Games–Howell). Profile labels are shorthand for the observed indicator configurations and are not claims about discrete motivational types. The profile solution was not validated in an independent sample, and likelihood-based enumeration tests (Lo–Mendell–Rubin adjusted likelihood ratio test; bootstrapped likelihood ratio test) were not computed. MEQ-18, 18-item Mindful Eating Questionnaire; MSPPA, Scale of Motivation for Participation in Physical Activity; SD, standard deviation.

The profiles differed in mindful eating in a facet-specific manner, mirroring the variable-level findings. Welch’s ANOVA indicated significant between-profile differences in MEQ-18 Total [*F*(2, 304.9) = 15.97, *p* < 0.001, *η*^2^ = 0.049] and in the Awareness facet [*F*(2, 311.3) = 16.01, *p* < 0.001, *η*^2^ = 0.049] but no difference in the Disinhibition facet [*F*(2, 310.8) = 0.26, *p* = 0.770, *η*^2^ = 0.001]. Awareness increased monotonically across profiles (Profile 1 M = 2.514, SD = 0.556; Profile 2 M = 2.746, SD = 0.536; Profile 3 M = 2.820, SD = 0.656). Games–Howell comparisons, reported in Table 7B, showed that the Amotivation-prone profile scored significantly lower on Awareness than both the Individually referenced profile (Hedges’ *g* = 0.42, *p* < 0.001) and the Broadly endorsed profile (*g* = 0.52, *p* < 0.001), whereas the latter two profiles did not differ from each other (*g* = 0.13, *p* = 0.526); the same ordering of profiles held for MEQ-18 Total. By contrast, no pair of profiles differed on Disinhibition (all |*g*| ≤ 0.08, all *p* ≥ 0.75). Thus, the awareness-dominant, disinhibition-null asymmetry observed in the variable-level regressions was reproduced at the person level: Students in the profile with the weakest and least differentiated motivational configuration reported lower awareness-based mindful eating, whereas the motivational configuration showed no association with disinhibition-based eating.

## 4 Discussion

This study investigated whether motivation for physical activity participation is associated with mindful eating among university students in Türkiye (*N* = 638), using the MSPPA and MEQ-18 within an SDT framework. The design is cross-sectional; the findings describe concurrent associations and cannot support causal, directional, or mechanistic inference. Two results were most salient and mapped directly onto the three variable-level prespecified predictions (H1, H2a, and H2b) tested in the adjusted multiple linear regression models. First, the predicted positive association between motivation for participation in physical activity and overall mindful eating (H1) was supported, as was the predicted positive association between motivation and the awareness-based facet of mindful eating (H2a), which was the stronger of the two. Second, and as predicted, motivation showed little association with the disinhibition-based facet (H2b); although this negligible association formally aligns with the prediction that the disinhibition association would be weak relative to awareness, it is interpreted here as measurement-attenuated rather than as a substantive null, given *α*₍Disinhibition₎ = 0.568. The association between motivation and awareness-based mindful eating is consistent with broader SDT-based evidence linking autonomous motivation to cross-domain self-regulatory profiles in older adult populations (Palombi et al., 2025).

The MSPPA performed well: Internal consistency was good for the total score (*α* = 0.858) and acceptable to excellent for the subscales (*α* = 0.763–0.900). For the MEQ-18, internal consistency was strong for Awareness (*α* = 0.835) but sub-optimal for Disinhibition (*α* = 0.568) and the total score (*α* = 0.622), which materially constrains the inferential weight that can be placed on findings involving the Disinhibition-facet. Harman’s single-factor EFA and the single-factor CFA did not support common method variance as a dominant driver of covariance. Overall, the psychometric evidence supports confident interpretation of MSPPA constructs and a more tentative interpretation of MEQ-18 Total and Disinhibition scores.

The asymmetric pattern across MEQ-18 facets is consist with SDT-based theoretical reasoning. Awareness is proximal to autonomous motivation through three mechanisms: shared recruitment of attentional control (Cano and Gomez-Baya, 2025; Teixeira et al., 2012), competence-supported interoceptive precision (Warren and Frame, 2025; Warren et al., 2017), and autonomy-supported value alignment that may extend deliberative attention from activity choice to food choice (Carraça et al., 2020; Kao et al., 2024). Disinhibition, by contrast, reflects failures of inhibitory control under cue reactivity, stress, and negative affect, processes governed by affect-regulation and habit systems that are more distally related to motivational quality (Warren and Frame, 2025; Warren et al., 2017). This theoretical mapping is consistent with synthesis work indicating that mindful eating Awareness, more than Disinhibition, is associated with healthier eating practices (Allameh et al., 2025; Hinton et al., 2024; Warren et al., 2017) and with evidence that Disinhibition is more strongly associated with stress and negative affect (Warren and Frame, 2025). The conceptual reframing of mindful eating as mindful eating behavior, with greater specificity regarding the behavioral regulation pathway (Mantzios, 2021; Hasan et al., 2025), further supports facet-specific theorizing.

Two interpretive caveats temper this reasoning; the first is measurement attenuation: Given *α*₍Disinhibition₎ = 0.568, the disattenuated upper bound for the motivation-disinhibition association could be meaningfully larger than the observed B ≈ 0, and a properly powered test of the prediction that motivation is only weakly related to disinhibition-based eating (H2b) would require a higher-reliability disinhibition measure (e.g., the Turkish Mindful Eating Behavior Scale; Dinç et al., 2025). The second is theoretical underdetermination: The cross-sectional design cannot distinguish between SDT-based shared self-regulatory accounts and simpler confounding by dispositional self-control or health consciousness.

The person-centered analysis adds a layer of evidence that the variable-level regressions cannot provide; rather than assuming that a single motivation-mindfulness slope applies uniformly across students, the latent profile analysis identified three qualitatively distinct motivational configurations. It showed that the awareness-dominant, disinhibition-null asymmetry recurs when students are grouped by their whole motivational profile. The convergence of the variable-centered and person-centered results is informative: It suggests that the association with awareness is unlikely to be an artifact of averaging across heterogeneous subgroups since the students with the most impoverished motivational configuration (Profile 1) were precisely those who reported the lowest awareness-based mindful eating, whereas no profile stood out for disinhibition. This person-level convergence is consistent with SDT reading that autonomous, value-aligned engagement in activity co-occurs with attentive, awareness-based eating rather than with the inhibitory-control processes implicated in disinhibition; because the design is cross-sectional, co-occurrence, and not influence in either direction, is what these data can establish. At the same time, the contribution is bounded by the same cross-sectional and measurement constraints noted above: The profiles describe co-occurring configurations at a single time point, not developmental trajectories, and the ceiling-level Individual Causes and Environmental Causes scores in the Broadly endorsed profile indicate limited upper-range discrimination of the MSPPA in this sample, so the modest separation between the Individually referenced and Broadly endorsed profiles on awareness should not be over-interpreted. The profiles are nonetheless more directly actionable than a regression coefficient: They suggest that the roughly two-fifths of students in the Amotivation-prone profile, rather than the student body as a whole, may be a useful priority group for integrated, autonomy-supportive supports. One qualification is fundamental to all of these readings: The profile solution was estimated and evaluated in a single sample and was not validated in an independent sample, so the profiles should be treated as an exploratory description of the present dataset rather than as an established typology, and the labels attached to them remain heuristic and largely data-driven.

To situate the present results within the Turkish evidence base, the most directly relevant previous study is summarized narratively here, rather than in a separate table. On the measurement side, an earlier Turkish adaptation of the longer 30-item MEQ in a university-student sample (Kömürcü Akik and Yiğit, 2023) reported the same Awareness-dominant reliability profile, with the awareness-type subscales outperforming the disinhibition-type subscales, a pattern carried forward into the abbreviated 18-item version.

Kaya et al. (2024) validated the Turkish MEQ-18 in an adult sample (*α*_Awareness = 0.843; *α*_Disinhibition = 0.789; two-factor CFA); the present sample reproduces the Awareness reliability (*α* = 0.835) but not the Disinhibition reliability (*α* = 0.568). Two subsequent Turkish instruments, namely, the Mindful Eating Behavior Scale (Dinç et al., 2025) and the Expanded Mindful Eating Scale (Doğan et al., 2025), show more stable disinhibition-facet reliability, and Koçyiğit et al. (2025) link mindful eating to health literacy in a Turkish clinical sample. On the physical activity motivation side, Orhan et al. (2024, 2025a, 2025b, 2026) have consistently reported across four Turkish adult and student samples that activity frequency and motivational profiles co-vary with healthier nutrition-related attitudes. Two convergent patterns therefore emerge from the Turkish literature and are reproduced in the present data: Turkish MEQ-type Disinhibition subscales tend to underperform their original validation reliability in new samples, whereas Awareness subscales remain consistently reliable; and activity-related motivational variables track attentive/healthier eating attitudes across Turkish populations.

The Turkish higher-education context has features that shape health behaviors in ways not necessarily captured by Western samples. Against this backdrop, present findings align with Turkish adult and student studies that have linked physical activity frequency with healthier nutrition attitudes and knowledge (Orhan et al., 2024, 2025a, 2025b, 2026) and with Turkish psychometric work showing that mindful eating awareness tends to yield higher reliability than disinhibition-related subscales across Turkish samples (Dinç et al., 2025; Doğan et al., 2025; Kaya et al., 2024; Koçyiğit et al., 2025). In the present sample, the MEQ-18 Disinhibition subscale (*α* = 0.568) fell well below the reliability reported in the original Turkish MEQ-18 validation (*α* = 0.789; Kaya et al., 2024), a gap consistent with broader Turkish psychometric evidence that MEQ-type disinhibition subscales tend to underperform outside their original validation samples. Taken together, the Turkish literature converges on an Awareness-dominant pattern, with awareness-type subscales showing both higher internal consistency and stronger associations with health-relevant outcomes than disinhibition-type subscales (Dinç et al., 2025; Doğan et al., 2025; Kaya et al., 2024; Koçyiğit et al., 2025); the present finding that motivation aligns with Awareness but not with Disinhibition fits squarely within that pattern and, interpreted against it, reinforces the measurement-attenuation reading of the Disinhibition null.

International comparisons with Mexican college samples have likewise shown awareness-dominant patterns of association with eating behavior (Lazarevich et al., 2025). Several features of Turkish higher education plausibly shape the patterns reported here. Many Turkish undergraduates may live with family or in university dormitories, where food provisioning, meal timing, and portioning can be externally structured, which may co-occur with lower disinhibition-driven intake even where motivational quality for activity is uneven. Campus activity environments are also heterogeneous, with some major-city universities typically providing formal fitness infrastructure while smaller-city campuses may rely more on informal or off-campus activities; this structural heterogeneity may plausibly co-vary with between-student variability in motivational quality for participation in physical activity, even though formal between-group comparisons across physical activity frequency categories were not undertaken in the present analyses. Religious and seasonal eating rhythms, such as Ramadan fasting and shared evening meals, constitute cultural scripts around food attention that may coincide with higher awareness-based mindful eating, independently of physical activity motivation. Gendered norms around physical activity and body image in Türkiye are also in active flux (Orhan et al., 2025a), which may be relevant to the small but consistent sex differences in environmental motivation and in mindful eating observed here. Together, these features argue that the motivation-awareness association documented here is theoretically plausible within, and partly specific to, the Turkish higher-education context.

The associations reported here are statistically significant and remain stable under HC3-robust, FDR-corrected analysis, but their magnitude should temper practical claims. Adjusted R^2^ values of 0.10–0.20 indicate that the full adjusted model (covariates plus MSPPA) accounts for a small-to-moderate fraction of interindividual variance in mindful eating, with motivation making a small but reliable independent contribution and leaving substantial variance potentially attributable to dietary context, affect, stress, cultural food norms, and unmeasured self-regulatory traits, including identity- and anxiety-related processes documented in young-adult and young-women populations (Smith et al., 2025; Wang et al., 2024). Expressed in standardized units, the *B* = 0.243 MSPPA-Awareness coefficient corresponds to a difference of roughly 0.29 SD in MEQ-18 Awareness between students who differ by 1 SD in MSPPA, representing a small-to-moderate difference by Cohen’s conventions that would not, on its own, be expected to correspond to clinically meaningful differences in eating behavior in the absence of concurrent, scaffolded intervention. Practical implications should therefore be framed as a rationale for integrated, low-intensity behavior change supports rather than as stand-alone interventions.

### 4.1 Limitations

The cross-sectional design precludes causal inference; physical activity frequency was operationalized as days/week and cannot be directly mapped to the WHO time-based recommendations; future work should use the Turkish IPAQ-SF or accelerometry/wearable device data. BMI was self-reported. The lower reliability of the MEQ-18 Disinhibition subscale (*α* = 0.568) likely attenuated associations, rendering the Disinhibition null underpowered against true effects of small magnitude. Associations should be re-tested with higher-reliability Turkish instruments. Additional limitations include the absence of measures of total caloric intake, macronutrient composition, and stress-related variables, which are known confounders of both physical activity motivation and eating behavior, particularly among student populations under elevated academic stress.

Although the sample is educationally homogeneous and dominated by undergraduates (91.7%), the small master’s and doctoral subgroups (6.3 and 2.0%, respectively) may contribute to the right tail of the age distribution; coefficients for the primary association were comparable across the three age strata, and the age × motivation interaction was non-significant (*p* = 0.618), but generalization to graduate-only populations should be made with caution. Participants were recruited via institutional email and social media; digitally engaged, health-interested students may therefore be over-represented, and common method variance cannot be ruled out. Although Harman’s test and the single-factor CFA did not support a dominant method factor, both diagnostics are limited in their ability to detect common method variance (Kock, 2015). Findings derive from a single-country Turkish sample; replication in probability-based, multi-country samples is needed. Beyond these design and measurement caveats, the proposed explanatory mechanisms, shared attentional control, competence-supported interoceptive precision, and autonomy-supported value alignment, were not directly measured in this study; the variable-level regressions and the person-centered latent profile analysis therefore document the predicted pattern of associations and its person-level configuration but cannot identify the intervening processes that produce it. A formal mediation test of these mechanisms would require primary measures of the candidate mediators (e.g., interoceptive awareness, executive attention, stress, and emotion regulation), which the present dataset does not contain; entering the MSPPA subscales as parallel mediators of their own total score would be structurally circular and was therefore not undertaken. Future research in similar cross-sectional samples could fruitfully employ cross-sectional (associative or “atemporal”) mediation models that include such measured mediators to clarify the explanatory pathways linking motivation for physical activity to the awareness- and disinhibition-based facets of mindful eating, while remaining within the inferential limits of cross-sectional data; established regression-based mediation frameworks (Hayes, 2022) are well suited to this aim, and the well-documented bias in cross-sectional indirect-effect estimates relative to longitudinal designs (Maxwell and Cole, 2007) should be acknowledged when interpreting such estimates. Three further constraints attach specifically to the person-centered analysis; first, the three-profile solution was estimated and evaluated within a single sample and was not validated in an independent sample; the profiles are therefore an exploratory description of this dataset rather than an established typology, and future research should test whether the same number, size, and shape of profiles are recovered in independent Turkish and non-Turkish student samples, and whether profile membership is stable over time. Second, profile enumeration relied on information criteria, entropy, profile size, and substantive interpretability, without likelihood-based tests of the *k*- versus (*k* – 1)-profile solution such as the Lo–Mendell–Rubin adjusted likelihood ratio test or the bootstrapped likelihood ratio test; the retained solution is therefore supported by convergent but not by formally inferential enumeration evidence. Third, the profile labels are heuristic summaries of the observed indicator configurations and are largely data-driven; they should not be read as evidence for discrete motivational types in the population, and the theoretical interpretation offered for them is correspondingly provisional.

### 4.2 Practical implications

From an applied standpoint, the findings support integrated university health promotion approaches that treat physical activity and eating as linked self-regulatory behaviors (Annesi, 2025; Prats-Arimon et al., 2024). Because motivation aligned most consistently with mindful eating Awareness, and because the practical effects are small to moderate, integrated low-intensity supports may offer the best return on investment. Programs may benefit from combining autonomy-supportive physical activity strategies with brief mindful eating skills and from leveraging the affective and self-efficacy benefits of regular physical exercise, both of which align with known psychosocial and motivational determinants of eating and exercise behavior in university and adolescent populations.

Translating these principles into the Turkish higher-education context yields four concrete recommendations. First, because many Turkish undergraduates eat in university dormitory cafeterias or subsidized refectories, low-cost mindful eating cues, pre-meal prompts on tray liners, digital signage at serving stations, and brief pauses in queue flow can be embedded into existing food-service infrastructure. Second, Ramadan and other culturally salient eating rhythms may provide opportunities for culturally tailored intervention: brief mindful eating modules delivered in the 2 weeks preceding Ramadan, paired with activity scheduling that accommodates fasting and evening routines, could leverage an already-established cultural context around deliberative eating. Third, uneven gendered access to campus fitness infrastructure can be narrowed through women-only activity windows, safe campus walking routes, and peer-led cohorts, addressing the environmental-motivation gap observed here between male and female students. Fourth, smaller-city campuses with limited on-campus facilities may benefit more from partnerships with outdoor and community-based activities than from investment in dedicated gyms, consistent with broader SDT-based evidence that environmental affordances supporting autonomous, intrinsically rewarding activity choices are associated with greater adherence than facility-centered provision alone (Teixeira et al., 2012).

## 5 Conclusion

In a sample of 638 university students in Türkiye, higher motivation to participate in physical activity was associated with greater mindful eating, most consistently for the Awareness facet, a pattern consistent with SDT-based reasoning. Psychometric indices supported the use of the MSPPA Total and subscales, whereas MEQ-18 internal consistency was acceptable for Awareness but low for Disinhibition and modest for the total score; the null association with Disinhibition should be interpreted as measurement attenuation rather than necessarily reflecting a substantive effect. In adjusted multiple linear regression models with HC3 robust standard errors and covariate adjustment for age, sex, and body mass index category, MSPPA Total remained positively associated with MEQ-18 Total and with the Awareness facet. Harman’s single-factor test and a single-factor CFA did not support common method bias as the principal driver of the observed associations, and coefficients for the primary association were comparable across the three age strata reflecting the program-level composition of the sample (age × motivation interaction, *p* = 0.618). A complementary person-centered latent profile analysis distinguished three motivational profiles that differed in awareness-based but not disinhibition-based mindful eating, reproducing the same facet-specific pattern at the person level and indicating that the profile with the weakest and least differentiated motivational configuration may be a useful priority group for tailored support. This profile solution was derived and evaluated within a single sample and was not validated in an independent sample, so it requires replication before the profiles are treated as an established typology. As the design is cross-sectional, the findings describe concurrent associations and do not establish directionality. Longitudinal and intervention studies using more reliable Turkish mindful eating measures, objective activity indicators (including IPAQ-SF and accelerometry), and comprehensive dietary assessment are needed to test directionality and identify mechanisms.

## Data Availability

The raw data supporting the conclusions of this article will be made available by the authors, without undue reservation.
